# Oxidative stress in the brain–lung crosstalk: cellular and molecular perspectives

**DOI:** 10.3389/fnagi.2024.1389454

**Published:** 2024-04-03

**Authors:** Jianda Kong, Rao Fan, Yuanqi Zhang, Zixuan Jia, Jing Zhang, Huixin Pan, Qinglu Wang

**Affiliations:** ^1^College of Sports Science, Qufu Normal University, Jining, China; ^2^College of Sport and Health, Shandong Sport University, Jinan, China

**Keywords:** oxidative stress, reactive oxygen species, brain, lung, brain–lung crosstalk, reactive nitrogen species, 8-hydroxy-2′-deoxyguanosine

## Abstract

Oxidative stress is caused by an imbalance between the production of reactive oxygen species (ROS) and the body’s ability to counteract their harmful effects, playing a key role in the pathogenesis of brain and lung-related diseases. This review comprehensively examines the intricate mechanisms by which oxidative stress influences cellular and molecular pathways, contributing to neurodegenerative, cardiovascular, and respiratory disorders. Emphasizing the detrimental effects on both brain and lung health, we discuss innovative diagnostic biomarkers, such as 8-hydroxy-2′-deoxyguanosine (8-OHdG), and the potential of antioxidant therapies. For these topics, we provide insights into future research directions in the field of oxidative stress treatment, including the development of personalized treatment approaches, the discovery and validation of novel biomarkers, and the development of new drug delivery systems. This review not only provides a new perspective on understanding the role of oxidative stress in brain and lung-related diseases but also offers new insights for future clinical treatments.

## 1 Introduction

Oxidative stress, characterized by the imbalance between the production of reactive oxygen and nitrogen species (ROS/RNS) and the detoxification capacity of the body toward these reactive intermediates, plays a crucial role in various diseases including the brain and lungs. In the cellular environment, oxidative stress disrupts cell signaling, gene expression, and protein function through the accumulation of ROS and RNS, leading to cell damage and death (Forman and Zhang, 2021; Jelinek et al., 2021). Under physiological conditions, ROS and RNS indeed play pivotal roles in cellular signaling, immune function, and the homeostasis of the cellular environment. They act both as signaling molecules that promote physiological responses and, when in excess, can lead to oxidative stress pathologies characterized by cellular and biochemical complexities (Zarkovic, 2020). However, an excess of ROS and RNS can indeed have deleterious effects, including the oxidative damage to DNA, proteins, and lipids, thereby compromising cellular function and integrity (Schieber and Chandel, 2014).

In the brain, oxidative stress is closely associated with a range of neurodegenerative diseases, stroke, and traumatic brain injury (TBI). Accumulation of ROS can result in neuronal damage and loss, making significant contributions to the pathophysiology of these diseases. Furthermore, oxidative stress is also linked to neuroinflammation and blood–brain barrier dysfunction, which are key features in the progression of neurological disorders (Jelinek et al., 2021). Accumulation of ROS in the brain can cause oxidative damage to DNA, proteins, and lipids, leading to neuronal injury and loss (Radak et al., 2011). In addition, oxidative stress is closely associated with neuroinflammation, which serves as a defensive but potentially harmful response of the brain’s immune system to various damages (Teleanu et al., 2022). Oxidative stress also disrupts the integrity of the blood–brain barrier, resulting in increased permeability and functional impairment. This disruption allows inflammatory cells and potentially harmful substances to infiltrate the brain, further contributing to neuronal damage and the progression of neurodegenerative diseases (Katsi et al., 2020).

Similarly, in the lungs, oxidative stress is a critical factor in the pathogenesis of diseases such as chronic obstructive pulmonary disease (COPD), pulmonary fibrosis, and acute respiratory distress syndrome (ARDS). Oxidative damage in lung tissue can lead to disruption of cellular barrier function, enhanced inflammatory response, and aggravated fibrosis process (Hecker, 2018; Bezerra et al., 2023). This pathological condition arises when there is an imbalance between the production of ROS and the lung’s ability to detoxify these harmful compounds or to repair the resulting damage (Snezhkina et al., 2019). Reactive oxygen species, such as free radicals and peroxides, are generated as byproducts of normal cellular metabolism; however, their levels can become excessively elevated due to environmental factors such as pollution, cigarette smoke, or infections (Sies and Jones, 2020).

Despite the anatomical and functional differences between the brain and lungs, they engage in complex bidirectional communication under conditions of oxidative stress. For instance, brain injury can activate the neuroimmune lung axis, potentially leading to pulmonary pathology (Ziaka and Exadaktylos, 2021). Conversely, lung diseases can impact neural function through oxidative stress and inflammatory pathways (Bezerra et al., 2023). These interactions emphasize the importance of considering the health of two organs in the context of a disease that may initially affect one organ, implying that when one organ experiences stress or damage, it can trigger a cascade of reactions in another organ.

Based on the information presented above, this review aims to delve into the molecular mechanisms of oxidative stress in the interplay between the brain and lungs, with a specific focus on dynamic changes under healthy and disease conditions. By synthesizing existing research, we aim to elucidate how oxidative stress affects the interaction between these two critical organ systems and its potential implications for future therapeutic strategies.

## 2 Basic concepts of oxidative stress

### 2.1 The generation and regulation of free radicals and ROS

Free radicals and ROS are molecules with at least one unpaired electron, making them highly reactive. These substances include oxygen free radicals (such as superoxide anion, hydroxyl radical, perhydroxyl radical, and singlet oxygen) as well as nitrogen free radicals. Under physiological conditions, ROS are produced in liver cells and macrophages through cellular processes such as aerobic respiration or inflammatory processes. ROS primarily function as signaling molecules and also participate in cell differentiation and apoptosis, thereby promoting the natural aging process (Jakubczyk et al., 2020). Excessive production of ROS may be caused by prolonged exposure to UV radiation, chronic stress, intense physical activity, improper diet, and the use of irritants (Lushchak, 2015).

### 2.2 The effects of oxidative stress on cell function

Reactive oxygen species are chemically reactive molecules containing oxygen that play a dual role within biological systems, acting both as vital signaling molecules and as detrimental agents when present in excess. They interact with several molecules within cells, significantly affecting cell function. Specifically, ROS can react with three major classes of macromolecules: lipids, proteins, and DNA.

Lipids are susceptible to ROS through a process known as lipid peroxidation, where ROS attack the polyunsaturated fatty acids in cell membranes (Kim et al., 2023). This attack leads to the formation of lipid peroxides, disrupting the membrane’s integrity and fluidity, and can result in cell lysis or apoptosis. Additionally, proteins can also be modified by ROS through oxidation of amino acid residues, particularly cysteine and methionine, leading to changes in protein structure and function (Kim et al., 2014). This modification can affect enzyme activity, receptor function, and signal transduction pathways, altering the cell’s normal operations and responses. DNA is another critical target of ROS. Oxidative damage to DNA includes base modifications, strand breaks, and cross-linking, which can result in mutations and chromosomal aberrations. These genetic alterations can disrupt normal cell cycle progression, affect gene expression, and lead to carcinogenesis (Lovell and Markesbery, 2007; Tung et al., 2012). Moreover, ROS influence cellular processes such as proliferation, differentiation, and apoptosis by modulating various signal transduction pathways and gene expression mechanisms. For instance, ROS can activate or inhibit transcription factors like NF-κB and AP-1, which are involved in the regulation of genes responsible for cell survival, growth, and death (Fujioka et al., 2004; Morgan and Liu, 2011). However, the accumulation of excessive ROS within cells can trigger cellular stress responses, such as the activation of antioxidant defense mechanisms or the induction of programmed cell death pathways. When the balance tips toward an overproduction of ROS, it can lead to oxidative stress, resulting in significant cell damage or death. This oxidative stress is a key pathogenic factor in the development of various diseases, including neurodegenerative disorders, cardiovascular diseases, and cancer (Schieber and Chandel, 2014).

In summary, ROS interact with and can damage key cellular molecules like lipids, proteins, and DNA, thereby disrupting normal cellular functions and contributing to disease pathogenesis. The body’s ability to counteract or repair such damage is crucial for maintaining cellular health and preventing disease progression. Table 1 demonstrates an overview of ROS interactions with cellular macromolecules and their biological roles.

**TABLE 1:** Reactive oxygen species interactions with cellular macromolecules and their biological effects.

Molecule class	Interaction with ROS	Effects	References
Lipids	ROS attack polyunsaturated fatty acids in cell membranes through lipid peroxidation.	Leads to the formation of lipid peroxides, disrupting membrane integrity and fluidity, potentially causing cell lysis or apoptosis.	Kim et al., 2023
Proteins	ROS modify proteins through oxidation of amino acid residues, particularly cysteine and methionine.	Changes in protein structure and function affecting enzyme activity, receptor function, and signal transduction pathways.	Kim et al., 2014
DNA	ROS cause oxidative damage to DNA, including base modifications, strand breaks, and cross-linking.	Lead to mutations and chromosomal aberrations, disrupting cell cycle, affecting gene expression, and potentially leading to carcinogenesis.	Tung et al., 2012
Signal transduction pathways	ROS modulate various signal transduction pathways and gene expression mechanisms, influencing cellular processes like proliferation, differentiation, and apoptosis.	Activation or inhibition of transcription factors such as NF-κB and AP-1, regulating genes responsible for cell survival, growth, and death.	Fujioka et al., 2004; Morgan and Liu, 2011

### 2.3 The association between oxidative stress and disease

A myriad of intricate connections between oxidative stress and diseases has been substantiated. Oxidative stress is a state that can lead to damage to cellular structure and function, characterized by the involvement of oxygen-containing free radicals capable of undergoing oxidation reactions. These radicals are produced during normal cellular metabolism and play crucial roles in vital physiological processes such as cell signaling and immune function (Chaudhary et al., 2023). However, when the accumulation of free radicals surpasses the scavenging capacity of antioxidants within the body, oxidative stress ensues, potentially leading to damage to proteins, lipids, and DNA, thereby precipitating the development of various diseases (Chaudhary et al., 2023). Consequently, a close association exists between oxidative stress and diseases related to the brain and lungs. For instance, an acute increase in ROS production following ischemic stroke overwhelms antioxidant defenses, leading to further tissue damage. Reperfusion therapy, despite facilitating blood reflow, results in the generation of highly detrimental ROS, culminating in oxidative stress that contributes to the majority of ischemic reperfusion injuries and, consequently, brain tissue damage (Allen and Bayraktutan, 2009). Furthermore, oxidative stress and inflammation play significant roles in both acute and chronic lung injury, where new therapeutic targets include mitochondrial ROS, NLRP3 inflammasomes, DNA sensors, cell death pathways, and IL-1 inhibitors (Wiegman et al., 2020).

In the progression of cancer, oxidative stress also plays a pivotal role. Oxidative stress can promote the onset and progression of tumors, including brain and lung cancers. Regarding brain cancer, oxidative stress can accelerate tumor cell proliferation and invasion through mechanisms such as DNA damage, promotion of inflammatory responses, and alteration of cell signaling pathways. For instance, glioblastoma multiforme (GBM), a highly aggressive form of brain cancer, is associated with oxidative stress-related DNA damage (Rezatabar et al., 2019). Moreover, by altering the tumor microenvironment, such as affecting angiogenesis around the tumor, oxidative stress further promotes the growth and metastasis of brain cancer (Aboelella et al., 2021). Similarly, oxidative stress plays a key role in lung cancer. Smoking, a major risk factor for lung cancer, produces a significant amount of free radicals, leading to oxidative stress. This not only directly damages the DNA of lung cells, increasing the risk of mutations and thus promoting carcinogenesis, but also activates inflammatory responses, facilitating the establishment of a tumor microenvironment conducive to the development of lung cancer (Caliri et al., 2021). Additionally, oxidative stress, including that mediated by the nicotinamide adenine dinucleotide phosphate (NADPH) oxidase family, can further promote cancer progression through mechanisms affecting apoptosis, cell cycle regulation, and intercellular signaling, potentially following the same mechanisms in lung cancer (Vermot et al., 2021).

## 3 Role of oxidative stress in the lung

### 3.1 The biological basis of oxidative stress in the lungs

In the pathophysiology of asthma, recruitment and activation of inflammatory cells such as neutrophils, macrophages, and eosinophils lead to increased levels of ROS and RNS, triggering oxidative/nitrosative stress and reducing antioxidant enzyme activity. This process activates transcription factors such as nuclear factor erythroid 2-related factor 2 (NRF2) and nuclear factor-kappa B (NF-κB). The pathophysiology of COPD is influenced by oxidative substances, resulting in chronic inflammation and the development of diseases such as emphysema and bronchitis. Gao et al. (2015) emphasized the decreased expression of Klotho in airway epithelial cells of COPD patients and its impact on inflammation and oxidative damage in their study. Sokolowska et al. (2019) investigated the effects of ozone exposure on the respiratory barrier in a mouse model, revealing how oxidative stress triggers inflammation and airway remodeling. Additionally, Wiegman et al. (2015) explored how oxidative stress-induced mitochondrial dysfunction drives inflammation and airway smooth muscle remodeling in COPD patients. Antioxidant mechanisms play a crucial role in the lung’s response to inflammation caused by tobacco smoke and other substances (Wiegman et al., 2015). In idiopathic pulmonary fibrosis (IPF), factors such as age and environmental exposure promote its development. Oxidative stress and inflammation play important roles in tissue repair and remodeling. Over-oxygenation of healthy lungs leads to injury and inflammation, exacerbating lung inflammation under existing conditions and activating pathways such as mitogen-activated protein kinase (MAPK), c-Jun N-terminal kinase (JNK), and NF-κB (Alharbi et al., 2022).

### 3.2 The role of oxidative stress in lung diseases

#### 3.2.1 COPD

Oxidative stress plays a crucial role in the pathogenesis of COPD. COPD is characterized by chronic airway inflammation, and oxidative stress exacerbates this process by promoting the activation of inflammatory cells and the release of inflammatory mediators. Accumulation of ROS is considered a key factor in airway remodeling in COPD, leading to epithelial cell damage, fibrosis, and airway smooth muscle proliferation (Rahman, 2005). In addition, oxidative stress is associated with systemic inflammation and lung function impairment in COPD patients (Nucera et al., 2022).

**FIGURE 1:**
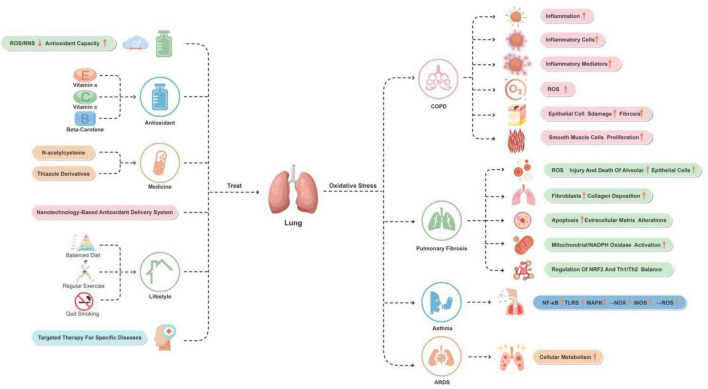
The role of oxidative stress in lung disease with associated and therapeutic strategies. This figure provides an overview of the oxidative stress mechanisms and improvement strategies for lung-related diseases and conditions. The major diseases and conditions include COPD, pulmonary fibrosis, asthma, and ARDS. By reducing ROS/RNS levels and increasing antioxidant capacity, using antioxidants and medications, and making lifestyle changes, oxidative stress in these diseases and conditions can effectively be reduced.

Long-term smoking is a major cause of COPD, providing an important source of chronic inhaled oxidants. Additionally, several inflammatory and structural cells in the lower airways of COPD patients act as endogenous sources of oxidants, even in ex-smokers (Mumby and Adcock, 2022). A systematic review have indicated that oxidative stress in the lower airways increases during stable and exacerbation periods in COPD patients compared to age-matched smokers (Zinellu et al., 2021). Therefore, antioxidant therapy strategies such as N-acetylcysteine and antioxidant supplementation have been shown to be beneficial in COPD patients, suggesting that reducing oxidative stress can improve disease symptoms and quality of life (Nucera et al., 2022). Figure 1 depicts these mechanisms.

#### 3.2.2 Pulmonary fibrosis

Pulmonary fibrosis is a disease characterized by abnormal repair and fibrosis of lung tissue, in which oxidative stress plays a crucial role in its development. During the process of pulmonary fibrosis, there is an increased production of ROS, resulting in damage and death of alveolar epithelial cells, subsequently triggering activation of fibroblasts and excessive deposition of collagen. This process involves various molecular mechanisms, including cell apoptosis and alteration of extracellular matrix. Studies have indicated that environmental toxins, activation of mitochondria/NADPH oxidase, and depletion of antioxidant defense are the main sources of oxidative stress in pulmonary fibrosis (Mastruzzo et al., 2002).

Furthermore, oxidative stress also promotes inflammation, exacerbating lung tissue damage and the fibrotic process. For instance, NRF2 plays a role in regulating oxidative stress levels in the lungs and T helper type 1/T helper type 2 (Th1/Th2) balance, which may have important implications in the development of pulmonary fibrosis (Walters et al., 2008).

**FIGURE 2:**
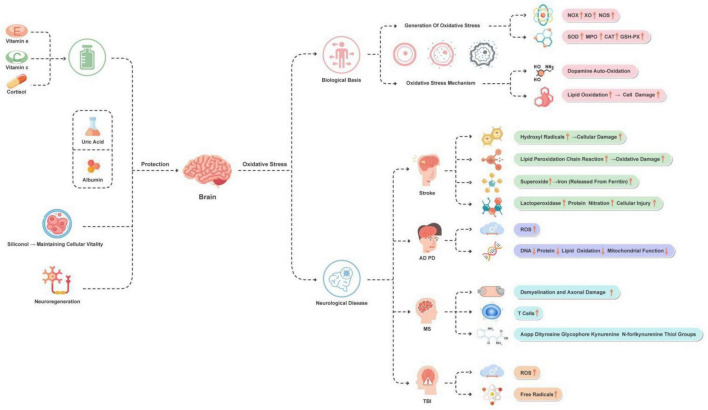
Main manifestations of oxidative stress in the brain and protection strategies. This figure illustrates the oxidative stress mechanisms and improvement strategies of brain-related diseases. The main diseases and conditions include stroke, AD, PD, MS, and TBI. The main improvement strategies involve the use of antioxidants, uric acid, albumin, silicic acid (for maintaining cell vitality), and promoting neural regeneration.

Antioxidant therapy has shown potential in managing pulmonary fibrosis. For example, the use of antioxidants such as N-acetylcysteine may help slow down the progression of the disease and improve lung function in patients. The effectiveness of this treatment approach may be attributed to its role in neutralizing ROS and enhancing antioxidant defense mechanisms (Walters et al., 2008). Figure 2 depicts these mechanisms.

#### 3.2.3 Other pulmonary diseases

In addition to COPD and pulmonary fibrosis, oxidative stress also plays a crucial role in other pulmonary diseases. The role of oxidative stress varies greatly among different pulmonary diseases. In the case of asthma, oxidative stress promotes its development by affecting redox-sensitive signaling pathways. Low levels of oxidative stress activate the Keap1-NRF2-ARE signaling pathway, inducing the expression of antioxidant and detoxification enzyme genes to eliminate excessive ROS (Liu K. et al., 2022). On the other hand, higher levels of oxidative stimulation activate the NF-κB, Toll-like receptors (TLRs), and MAPK signaling pathways, leading to the upregulation of inflammatory mediators, including pro-inflammatory cytokines and pro-oxidant enzymes, such as NADPH oxidase (NOX) and inducible nitric oxide synthase (iNOS), resulting in excessive ROS production. Additionally, TLRs also promote increased ROS production by mitochondria (Liu K. et al., 2022).

In the pathogenesis of asthma, the imbalance between excessive ROS production and reduced antioxidant defense mechanisms leads to the generation of oxidative stress. Oxidative stress plays an important role in the development and progression of asthma, which is characterized by the accumulation of inflammation and immune events. In asthma, the generation of oxidative stress occurs due to the imbalance between excessive ROS production and reduced antioxidant defense mechanisms (Liu K. et al., 2022; Michaeloudes et al., 2022).

In addition to the well-established mechanisms of oxidative stress in asthma, recent studies have expanded our understanding of nitrosative stress and its biomarkers in other pulmonary conditions. For instance, Galiniak et al. (2023) explored biomarkers of nitrosative stress in cystic fibrosis, revealing significant findings in the serum of patients, which underscores the complex interplay between oxidative and nitrosative stress in pulmonary diseases. Another study highlights the critical roles of oxidative stress in pulmonary disease progression, further emphasizing the need for detailed investigations into these mechanisms across a range of conditions (Pinzaru et al., 2023).

In ARDS, oxidative stress has been studied from a multidisciplinary perspective regarding its role in cellular metabolism in lung health and disease. For instance, a study investigated the effects of *Taxus cuspidata* ethanol extract (TO) and TO-mediated photodynamic therapy (TO-PDT) on A549 lung cancer cells. Morphological changes in the cell nucleus and cell membrane were observed in cells treated with TO and TO-PDT. These findings suggest that TO can serve as a photosensitizer during PDT to enhance its direct cytotoxic effects on cancer cells (Arunachalam et al., 2022; Liu K. et al., 2022).

### 3.3 Therapeutic strategies for oxidative stress

In the treatment of oxidative stress-related diseases, such as brain and lung diseases, therapeutic strategies indeed involve reducing the generation of ROS/RNS and enhancing the body’s antioxidant defense capacity. These diseases include neurodegenerative diseases, cardiovascular diseases, and cancer, among others. In this regard, traditional antioxidants, such as vitamin E, vitamin C, and beta-carotene, have been used to neutralize free radicals in the body and reduce oxidative stress. These antioxidants lower the levels of ROS/RNS by directly reacting with free radicals, thereby protecting cells from oxidative damage (Firuzi et al., 2011). Certain drugs, such as n-acetylcysteine (NAC) (Zhang et al., 2011)and thiazolidine derivatives (TZDs) (Sá et al., 2017), can increase the intracellular levels of glutathione, an important intracellular antioxidant that helps neutralize ROS (Firuzi et al., 2011).

Recent research has shown that antioxidant delivery systems based on nanotechnology are being studied to more effectively deliver antioxidants to cells and tissues affected by oxidative stress. These systems enhance treatment efficacy by improving the bioavailability and targeting of the drugs. For instance, antioxidant-based nanotherapy has demonstrated advantages in alleviating oxidative stress in neurodegenerative diseases, effectively neutralizing oxidative stress by improving the half-life and bioavailability of antioxidants and enhancing their ability to cross the blood–brain barrier (Ashok et al., 2022).

In addition, lifestyle improvements such as a balanced diet, regular exercise, and avoiding smoking are also considered effective means of reducing oxidative stress. These changes help enhance the natural antioxidant defense system and reduce the risk of chronic diseases caused by oxidative stress (Sorriento et al., 2018). Targeted treatments for specific diseases, such as alleviating the condition of COPD or neurodegenerative diseases using certain medications, may also involve reducing oxidative stress. These therapies may include specific antioxidants or anti-inflammatory drugs (Firuzi et al., 2011; Gonçalves and Romeiro, 2019). These are briefly depicted in Figure 1.

## 4 Oxidative stress in the brain

### 4.1 Biological basis of brain oxidative stress

In the brain, oxidative stress is a condition caused by an excessive production of free radicals and ROS, which are effectively neutralized under normal physiological conditions through antioxidant mechanisms. When this balance is disrupted, oxidative stress occurs in the brain. Enzymes such as NOX, xanthine oxidase (XO), nitric oxide synthase (NOS), superoxide dismutase (SOD), myeloperoxidase (MPO), catalase (CAT), and glutathione peroxidase (GSH-Px) are major contributors to oxidative stress (Jelinek et al., 2021).

Oxidative stress in the brain involves multiple mechanisms, including neurotransmitter auto-oxidation, lipid peroxidation, and redox signaling. For example, dopamine auto-oxidation produces semiquinone radicals, which further generate superoxide anions and hydrogen peroxide. Lipid peroxidation involves a multistep process, including generation of free radicals, oxidation, and propagation of chain reactions, all of which can lead to cell damage in the brain (Cobley et al., 2018).

Furthermore, oxidative stress in the brain is closely associated with neurofunction, pathological changes, and aging. Redox biology plays a crucial role in neurofunction, dysfunction, and aging. Studies have shown that redox signaling plays an important role in neurotransmitter release, cognitive function, and aging processes (Franco and Vargas, 2018).

### 4.2 Oxidative stress in neurological disorders

#### 4.2.1 Stroke

Ischemic stroke is a severe neurological disorder, and its pathogenesis is mainly related to oxidative stress. Oxidative stress plays a crucial role in the occurrence of stroke, directly affecting the severity of the condition and the recovery process of function.

Hydroxyl radicals are potent oxidants that rapidly oxidize neighboring molecules, leading to cell damage. Lipid peroxidation chain reactions are important oxidative stress reactions, which are initiated by hydroxyl radicals (Lushchak, 2014). Superoxide not only directly causes oxidative stress reactions but also acts as a precursor for hydrogen peroxide and peroxynitrite, further exacerbating the extent of oxidative damage (Quijano et al., 2007). Lipid peroxidation reactions result in damage to the brain cell membrane and play a vital role in the stroke process, intensifying cell injury (Manzanero et al., 2013). Additionally, superoxide promotes the release of iron from iron-sulfur proteins, catalyzing the production of more oxidative stress reactions (Kurtz, 2004). Peroxynitrite is a non-radical strong oxidant that can cause protein nitration, altering its function and leading to cell damage (Pisoschi and Pop, 2015).

All these mechanisms collectively contribute to the aggravation of oxidative stress reactions in stroke, resulting in neuronal damage and death. Studies have shown that the levels of malondialdehyde (MDA), a biomarker of lipid peroxidation, are related to the severity of stroke and functional recovery. Mendioroz et al. (2011) found that 59.9% of stroke patients showed a moderate to severe NIHSS score (16–20) at admission, while Mansour et al. (2015) reported a median NIHSS score of 22 (16–30) within 24 h and 20 (11–30) after 72 h (Elsayed et al., 2020). Moreover, the generation of superoxide and the formation of peroxynitrite are the main sources of oxidative stress in stroke. Studies have indicated that peroxynitrite, the non-radical form of peroxynitrite, is a very strong oxidant with reactivity of its decomposition intermediate, capable of rapidly causing protein nitration and altering protein function (Forman and Zhang, 2021).

Overall, these mechanisms collectively contribute to the exacerbation of oxidative stress during the stroke process, thereby affecting the pathophysiology of stroke and the recovery of patients. These aspects are all reflected in Figure 2.

#### 4.2.2 Neurodegenerative diseases

Neurodegenerative diseases such as Alzheimer’s disease (AD), Parkinson’s disease (PD), Huntington’s disease (HD), and multiple sclerosis (MS) exhibit significant effects of oxidative stress throughout their progression.

Reactive oxygen species production is a crucial process in neurodegenerative diseases. These ROS, including superoxide anions, hydroxyl radicals, hydroxide ions, and hydrogen peroxide, are the main mediators of oxidative stress (Kim et al., 2015). Oxidative stress leads to the oxidation of nucleic acids, proteins, and lipids, resulting in the formation of advanced glycation end products, impairment of mitochondrial function, activation of glial cells, deposition of amyloid-β plaques, cell apoptosis, cytokine production and inflammatory response, as well as dysfunction of proteasomes (Yaribeygi et al., 2018). Oxidative stress is considered a major factor contributing to neurodegeneration and cell death in AD, PD, and other neurodegenerative diseases. These diseases typically manifest as progressive loss of neurons and impaired motor or cognitive function (Niedzielska et al., 2016). Mitochondrial dysfunction is closely associated with oxidative stress, especially in the context of neurodegenerative diseases. Mitochondrial damage leads to cellular metabolic imbalance and reduced energy production, further exacerbating oxidative stress (Liu et al., 2017). Intriguingly, research into MS has revealed the pivotal role of oxidative stress in its pathogenesis. One article has demonstrated that ROS play a significant role in the etiology of MS, leading to demyelination and axonal damage, as well as the activation and proliferation of T cells (Ohl et al., 2016). Furthermore, an increase in oxidative and glycoxidative damage to serum proteins in MS patients has been identified, offering potential biomarkers for monitoring therapeutic efficacy and understanding the role of oxidative stress in the pathophysiology of MS. These biomarkers include protein carbonyls, advanced oxidation protein products (AOPP), dityrosine, glycophore, kynurenine, N′-formylkynurenine, and thiol groups, among others (Sadowska-Bartosz et al., 2013).

Due to the key role of oxidative stress in neurodegenerative diseases, antioxidant therapeutic strategies are considered potential approaches for these diseases. However, the effectiveness of these strategies is still controversial, and further research is needed to clarify their role in the treatment of neurodegenerative diseases. The above content is briefly outlined in Figure 2.

#### 4.2.3 TBI

In recent years, oxidative stress has increasingly been acknowledged as a key factor in the pathophysiology of TBI. Research has revealed the potential value of antioxidant therapy in mitigating the impact of TBI, particularly in combating the excessive generation of ROS and free radicals induced by brain injury (Hall et al., 2010). Additionally, mitochondrial dysfunction plays a significant role in the degradation of neuronal cytoskeleton following TBI, suggesting that targeting the mitochondrial pathway may be an effective intervention (Hill et al., 2017).

On the other hand, the administration of high-dose intravenous vitamin C as a novel approach for treating TBI has garnered considerable attention due to its demonstrated effectiveness in reducing oxidative stress and improving clinical outcomes (Leichtle et al., 2020). Furthermore, the development of novel antioxidant neuroprotective drugs has emerged as a crucial area of investigation, targeting specifically the oxidative stress pathway with the aim of protecting neurons from damage (Hall et al., 2019).

To comprehensively understand the impact of antioxidant therapy on TBI cases, a series of systematic reviews have been conducted. These evaluations extensively analyze how oxidative stress affects TBI patients and explore the potential benefits of antioxidant therapy (Shen et al., 2016). Moreover, research has identified an increased expression of mitochondrial deubiquitinase ubiquitin-specific protease 30 (USP30) following TBI, leading to impaired mitochondrial quality control. This finding suggests that targeting USP30 may be a novel strategy for alleviating secondary brain injury after TBI (Wu et al., 2023).

Overall, these studies collectively underscore the importance of addressing oxidative stress in the treatment of TBI and highlight the necessity of further exploring targeted therapeutic approaches to mitigate oxidative stress-induced damage to the brain. Therefore, oxidative stress holds crucial significance in the advancement of TBI research. Figure 2 reflects the above content.

### 4.3 Neuroprotection strategies

When oxidative stress damages the nervous system, various intervention strategies have shown potential effects. The application of antioxidants is considered a key approach. Studies have found a strong association between intake of vitamins C and E and delayed onset of AD in elderly individuals (Kontush and Schekatolina, 2004; Costanzi et al., 2008). Free radicals trigger neurodegenerative diseases through mechanisms such as lipid peroxidation, protein oxidation, and DNA damage (Franzoni et al., 2021). Kim et al.’s (2015) research further demonstrated that ROS cause cellular damage by disrupting the structure and function of biomolecules. Additionally, natural compounds such as quercetin possess neuroprotective properties. Arredondo et al.’s (2010) study revealed that quercetin can protect against oxidative stress-induced neuronal damage by promoting NRF2 nuclear translocation and increasing glutathione levels.

In the treatment of ischemic stroke, antioxidants such as uric acid and albumin have been proven to reduce stroke risk, with mechanisms involving the clearance of harmful ROS (Lee et al., 2020). Furthermore, maintaining cellular vitality during hypoxia is critical for neuroprotection strategies. Chtourou et al.’s (2012) research found that silibinin has a protective effect against manganese-induced neurotoxicity, achieved through improvements in cerebellar redox state and cholinergic functioning. Finally, significant progress has been made in research on neural regeneration in the field of neurodegenerative diseases. Despite the limited self-healing and regenerative capabilities in the central nervous system, current studies are exploring regenerative therapeutic approaches for neurodegenerative diseases (Simon and Standaert, 1999).

These research directions and findings provide a series of potential neuroprotection strategies, highlighting the potential value of antioxidants and natural compounds, as well as revealing new treatment targets and methods. These achievements lay a solid foundation for future research and clinical practice. Figure 2 depicts these treatment strategies.

## 5 Oxidative stress in brain–lung crosstalk

### 5.1 How oxidative stress impacts brain–lung communication

The significant role of oxidative stress in lung diseases has been established through research, particularly in the context of aging and chronic diseases such as COPD. Studies have highlighted the impact of cellular aging on respiratory tract infections and emphasized the importance of understanding plasma membrane repair mechanisms in pulmonary diseases (Cong et al., 2017; Yanagi et al., 2017). In addition, Cho and Stout-Delgado (2020) stressed the influence of aging on lung diseases, with oxidative stress potentially exacerbating this impact. Extensive literature supports the involvement of oxidative stress in neurodegenerative diseases. It contributes to the pathophysiology of conditions like AD, PD, and amyotrophic lateral sclerosis, promoting an imbalance in dopamine, iron, and alpha-synuclein in the brain, thereby generating oxidative stress (Bedard and Krause, 2007; Feitosa et al., 2018).

Research on oxidative stress genes associated with acute lung injury (ALI) has unveiled crucial biomarkers, highlighting the close relationship between oxidative stress and ALI and indicating the intertwining of immune response and oxidative stress in pulmonary diseases (Liu et al., 2023). The NOX, which generate ROS, play a significant role under both physiological and pathological conditions. Understanding the dynamics of these enzymes is essential for managing oxidative stress-related diseases affecting both the brain and lungs (Begum et al., 2022).

Oxidative stress-mediated disruption of the blood–brain barrier is a critical factor in neurological disorders. Iron metabolism, closely linked to oxidative stress, plays a crucial role in various neurological conditions. This aspect of oxidative stress has been demonstrated to impact diseases such as HD, PD, and AD (Song et al., 2020).

Thus, the relationship between oxidative stress and brain–lung crosstalk is intricate and multifaceted, involving a range of cellular and molecular processes. These studies provide a foundation for further exploration of oxidative stress-targeted therapeutic strategies in neurodegenerative and pulmonary diseases.

### 5.2 The role of ROS in brain–lung crosstalk: bridging neural, immune, and endocrine pathways

This section delves into the pivotal role of ROS in mediating brain–lung crosstalk through neural, immune, and endocrine pathways (Table 2). It illustrates how severe TBI can trigger pulmonary dysfunction via neuro-immune interactions facilitated by ROS, which disrupt the blood–brain barrier and enhance systemic inflammation, leading to conditions like ARDS. Furthermore, the immune pathways highlight ROS’s critical function in activating brain’s immune cells and macrophages, contributing to inflammation and influencing lung health. This is further compounded by endocrine pathways, where the hypothalamic-pituitary-adrenal (HPA) axis’s regulation by oxidative stress affects immune responses and susceptibility to lung infections and inflammation. Additionally, at a molecular level, ROS influence key signaling pathways such as NF-κB and MAPK, crucial for regulating inflammation and cellular stress responses, thus underscoring ROS’s comprehensive role in bridging neural, immune, and endocrine mechanisms affecting both brain and lung health.

**TABLE 2:** Detailed analysis of the role of ROS in brain–lung crosstalk: bridging neural, immune, and endocrine pathways.

Section	Description	Key mechanisms	References
Neural pathways	Explains how severe TBI induces pulmonary dysfunction through neuro-immune interactions mediated by ROS. This leads to blood–brain barrier disruption, systemic inflammatory mediator spread, and conditions like ARDS.	Neuro-immune interactions Blood–brain barrier disruption	Qian et al., 2020
Immune pathways	Highlights ROS’s role in activating brain’s immune cells and macrophages, contributing to inflammation and lung health. Discusses ROS’s involvement in immune response, metabolic reprogramming, and redox factors.	Activation of microglia and macrophages Metabolic reprogramming and redox factors in immune response	Yang et al., 2013; Banerjee et al., 2020; Morris et al., 2022
Endocrine pathways	Discusses the impact of oxidative stress and the HPA axis on immune function and lung susceptibility to infections and inflammation. Highlights the regulation of oxidative stress by ROS and its implications for health.	HPA axis regulation Oxidative stress mechanisms in immune function	Spiers et al., 2014; Leistner and Menke, 2020; Palma-Gudiel et al., 2021
Molecular and cellular mechanisms	Details how ROS affect key signaling pathways such as NF-κB and MAPK, crucial for inflammation and cellular stress response regulation. Also discusses the role of enzymes like NADPH oxidase in ROS production and cellular defense.	NF-κB and MAPK signaling pathways Enzymatic production of ROS and cellular defense	Morgan and Liu, 2011; Wang et al., 2017; Iqbal et al., 2024

#### 5.2.1 Neural pathways

Neurological injuries, particularly severe TBI, have been shown to induce pulmonary dysfunction through neuro-immune interactions. These interactions are mediated by ROS, which exacerbate lung inflammation by disrupting the blood–brain barrier, facilitating the systemic spread of inflammatory mediators. For instance, a study demonstrated that TBI can lead to ARDS, where ROS play a significant role in the pathogenesis by promoting inflammation and exacerbating lung damage (Qian et al., 2020).

#### 5.2.2 Immune pathways

The immune response plays a crucial role in bridging brain and lung health, significantly influenced by ROS. ROS are highly reactive chemicals containing oxygen, produced either exogenously or endogenously, and are essential for various biological functions, including cell survival, proliferation, differentiation, and immune response. They are related to a wide variety of human disorders, such as chronic inflammation, age-related diseases, and cancers (Yang et al., 2013).

In the context of neuroinflammation, ROS activate microglia, the resident immune cells of the brain, leading to the production of inflammatory cytokines. This process exacerbates neuroinflammation and can affect pulmonary health by inducing a systemic inflammatory response (Morris et al., 2022). The activation of macrophages by ROS involves metabolic reprogramming and redox factors, leading to the activation of proinflammatory cytokines and chemokines, iNOS, and HIF1α, crucial for maintaining macrophage activation and M1 polarization (Morris et al., 2022). This shift is indispensable for increasing phagocytosis, production of ROS, and proinflammatory cytokines. Conversely, pulmonary infections increase ROS production in lung tissue, affecting the central nervous system by enhancing neuroinflammation through cytokine-mediated mechanisms.

Reactive oxygen species-associated immune response and metabolism are intricately linked to the pathophysiology of various diseases, highlighting the role of metabolic reprogramming in immune responses, particularly in macrophages (Banerjee et al., 2020). The regulation of oxidative stress by ROS is crucial for both initiating and resolving inflammation, underlining the complex role ROS play in immune responses and disease pathogenesis.

#### 5.2.3 Endocrine pathways

Endocrine interactions, particularly those involving oxidative stress and the brain–lung crosstalk, are indeed complex and significantly influenced by the HPA axis. The regulation of the HPA axis and its interaction with oxidative stress mechanisms have profound implications for immune function and susceptibility to infections and inflammation in the lungs (Spiers et al., 2014; Leistner and Menke, 2020; Palma-Gudiel et al., 2021).

A study highlighted that HPA axis dysregulation is associated with altered immune function through epigenetic mechanisms, including DNA methylation changes in genes related to immune and HPA axis function. It found that higher cortisol levels, indicative of blunted HPA axis feedback, were associated with lower DNA methylation at certain gene sites involved in immune response regulation, suggesting a link between chronic stress, HPA axis function, and immune response modulation (Palma-Gudiel et al., 2021). Further research into the HPA axis’s role in stress responses elucidates how its dysregulation can contribute to various mental and physical health issues (Leistner and Menke, 2020). This research has identified distinct sex differences in the HPA axis’s regulation in response to stress, which might explain the variations in susceptibility to stress-related disorders between genders (Leistner and Menke, 2020). Moreover, the activation of the HPA axis and subsequent release of glucocorticoids have been shown to induce oxidative stress within cells, particularly affecting the redox system (Spiers et al., 2014). This system plays a crucial role in managing oxidative phosphorylation and maintaining cellular health. The exposure to glucocorticoids, particularly in the hippocampus, has been linked to increased oxidative stress, highlighting the intricate relationship between HPA axis activity, glucocorticoid release, and cellular oxidative balance (Spiers et al., 2014).

These findings collectively underscore the intricate interplay between the HPA axis, endocrine responses, oxidative stress, and immune function. Understanding these relationships is crucial for developing interventions aimed at mitigating the adverse effects of stress and oxidative stress on health, particularly in the context of lung health and susceptibility to infections and inflammation.

#### 5.2.4 Molecular and cellular mechanisms

At the molecular level, ROS influence several key signaling pathways, including NF-κB and MAPK pathways, which play a significant role in the regulation of inflammation and cellular stress responses in various diseases affecting the brain and lungs. The activation of these pathways by ROS can lead to an increased expression of inflammatory cytokines, chemokines, and adhesion molecules, thereby promoting the recruitment of immune cells to sites of injury or infection.

Reactive oxygen species are produced by a variety of enzymes, including NADPH oxidase, which is crucial for cellular defense mechanisms. However, excessive ROS can lead to oxidative stress, damaging intracellular proteins, lipids, and nucleic acids. In response to ROS, cells may activate pathways such as NF-κB, promoting survival by increasing the expression of antioxidant proteins and preventing excessive inflammation and cellular damage (Morgan and Liu, 2011). Furthermore, the MAPK pathway is another key signaling cascade activated by ROS, leading to the phosphorylation and activation of transcription factors such as AP-1 and NF-κB. These factors regulate the expression of genes involved in cell survival, proliferation, and apoptosis, highlighting the complex balance between ROS production and cellular signaling mechanisms (Iqbal et al., 2024). Specifically, in lung diseases, urban particulate matter has been demonstrated to trigger lung inflammation through the activation of ROS-mediated MAPK and NF-κB signaling pathways, emphasizing the molecular mechanisms by which environmental pollutants can exacerbate inflammatory responses in respiratory diseases (Wang et al., 2017).

These findings collectively underscore the complex interplay between ROS and key cellular signaling pathways in regulating inflammation and stress responses, elucidating potential therapeutic targets for diseases characterized by aberrant inflammation and oxidative stress.

### 5.3 Oxidative stress and glycation in brain–lung crosstalk

Recent studies have underscored the intricate relationship between oxidative stress and glycation, notably through the generation of advanced glycation end products (AGEs) and their impact on brain–lung interactions. Oxidative stress, characterized by an imbalance between the production of ROS and antioxidant defense mechanisms, plays a pivotal role in initiating and accelerating the glycation process. Glycation, a non-enzymatic reaction where reducing sugars react with proteins, lipids, and nucleic acids, leads to the formation of AGEs. These AGEs accumulate in various tissues, including the brain and lungs, causing cellular dysfunction and disease progression (Xu et al., 2023).

In the context of brain–lung interactions, the accumulation of AGEs has been demonstrated to exacerbate oxidative stress, thereby creating a vicious cycle that promotes neurodegeneration and the development and progression of pulmonary diseases. For instance, AGEs can compromise the blood–brain barrier, leading to increased neuronal vulnerability and inflammation, hallmark features of several brain disorders (Dobi et al., 2021). Similarly, in the lungs, AGEs contribute to the pathogenesis of COPD and pulmonary fibrosis through the enhancement of oxidative stress and inflammatory responses (Otoupalova et al., 2020). Furthermore, the receptor for AGEs (RAGE) mediates many of the deleterious effects of AGEs in both the brain and lungs. Specifically, AGEs, through their interaction with RAGE, trigger signaling pathways that further promote oxidative stress, inflammation, and cellular damage (Tóbon-Velasco et al., 2014). This interaction underscores the critical role of the oxidative stress-glycation axis in regulating brain–lung interactions and highlights the potential of therapeutic interventions targeting this pathway. Strategies aimed at reducing AGE accumulation, inhibiting RAGE signaling, or enhancing antioxidant defense systems offer promising avenues for mitigating the adverse effects of oxidative stress and glycation in brain–lung interactions. Antioxidant therapies, AGE breakers, and RAGE antagonists represent some of the interventions currently under investigation, holding the potential to disrupt this harmful cycle and improve disease outcomes (Firuzi et al., 2011).

In summary, the interplay between oxidative stress and glycation represents a key mechanism influencing the pathophysiology of brain and lung diseases. Further understanding of this relationship and the development of targeted therapeutic strategies may significantly impact the management and treatment of conditions associated with oxidative stress and the accumulation of AGEs.

## 6 In-depth exploration of molecular mechanisms

In this chapter, we embark on a detailed exploration of the complex molecular mechanisms involved in oxidative stress, focusing on two key areas: the role of essential molecular pathways (see section “6.1 Role of key molecular pathways”) and the potential of biomarkers in biomedical research (see section “6.2 The potential of biomarkers in biomedical research”). In section “6.1 Role of key molecular pathways,” we delve into several pivotal molecular pathways such as NRF2-ARE, NF-κB, and MAPK pathways. These pathways play crucial roles in regulating the oxidative stress response. By examining their specific functions in brain and lung diseases, we gain insights into the pathogenesis of these conditions and identify important molecular targets for potential therapeutic strategies.

Following this, section “6.2 The potential of biomarkers in biomedical research” explores the significance of biomarkers in identifying and quantifying levels of oxidative stress. This section emphasizes the role of oxidative stress biomarkers in various diseases, particularly their applications in brain and lung disorders. Identifying these biomarkers allows researchers to more accurately monitor oxidative stress levels, thereby facilitating a more effective assessment of disease states and treatment outcomes.

Together, these two sections form the foundation for an in-depth exploration of the molecular mechanisms of oxidative stress, offering new perspectives for understanding and combating brain and lung diseases at the molecular level. Table 3 shows comparative overview of key molecular pathways and biomarkers of oxidative stress in various diseases.

**TABLE 3:** Comparative overview of key molecular pathways and biomarkers of oxidative stress in various diseases.

Disease	Key molecular pathways	Biomarkers	Level changes	Significance	References
Cerebral injury	NRF2-ARE (regulates genes for antioxidation and detoxification)	Mitochondrial oxidative stress indicators, 8-OHdG	Post-injury increase in oxidative markers and 8-OHdG	Indicates oxidative stress damage and efficacy of antioxidative therapies	Yan et al., 2008
Pulmonary conditions	NRF2 (reduces pulmonary tissue damage from ROS)	AOPPs, Ox-LDL, gene expression of detoxifying enzymes	Elevated AOPPs, Ox-LDL during oxidative stress	Reflects ROS impact and NRF2 activation effectiveness	Pall and Levine, 2015
Neurodegenerative diseases (AD and PD)	NF-κB (linked to oxidative stress-induced brain injuries)	8,12-Iso-PGF2α, oxidized lipids, nitrotyrosine, MDA	Increased lipid peroxidation markers (8,12-iso-PGF2α, MDA) and protein nitration	Markers of inflammation, cellular damage in neurodegeneration	Mythri et al., 2011; Butterfield, 2020
Pulmonary fibrosis	NF-κB (modulates oxidative stress-related inflammatory responses)	Ox-LDL, AOPP levels, 8-OHdG, MDA	Elevated AOPP, Ox-LDL, 8-OHdG, and MDA levels	Indicative of oxidative stress and inflammation in lung tissue	Shen et al., 2013; Gao et al., 2021
General oxidative stress	MAPK pathway, HO-1, SOD	8,12-Iso-PGF2α, 8-OHdG, 8-OHdA, oxidized lipids, nitrotyrosine	Varies with oxidative stress levels	General indicators of oxidative stress and damage	Maselli et al., 2002; Méthy et al., 2004; Ryter and Choi, 2005; Fan et al., 2019; Rezatabar et al., 2019

### 6.1 Role of key molecular pathways

Central to the oxidative stress response is the NRF2-ARE pathway. NRF2, a pivotal transcription factor in combating oxidative stress, orchestrates cellular defense against ROS-induced harm by regulating the expression of genes responsible for antioxidation and detoxification. Within the cerebral context, activation of the NRF2-ARE pathway fortifies the antioxidative defense mechanism, attenuating neuronal injury caused by oxidative stress. Studies reveal that activation of the NRF2-ARE pathway post-cerebral injury diminishes mitochondrial oxidative stress triggered by singlet oxygen and superoxide radicals, thereby aiding in the recuperation of cognitive functions (Yan et al., 2008). In pulmonary conditions, NRF2 emerges as a critical defensive element, lessening pulmonary tissue damage from ROS by enhancing the expression of genes for detoxifying enzymes responsive to oxidative stress (Pall and Levine, 2015).

Another integral molecular pathway is the NF-κB pathway. NF-κB, a transcription factor, assumes a pivotal role in the oxidative stress response. In the brain, oxidative stress catalyzes NF-κB activation, leading to escalated production of inflammatory mediators and increased neuronal apoptosis. Investigations have demonstrated a correlation between cerebral oxidative stress and the NF-κB pathway, suggesting that inhibiting this pathway may alleviate brain injuries induced by oxidative stress (Chen et al., 2021). In pulmonary diseases, activation of the NF-κB pathway is thought to play a role in modulating oxidative stress-related inflammatory responses. Studies indicate that suppressing NF-κB pathway activity can reduce oxidative stress and inflammatory reactions in pulmonary fibrosis, thereby promoting repair of lung tissue (Lingappan, 2018).

In addition to the NRF2-ARE and NF-κB pathways, other critical molecular pathways also partake in the regulation of oxidative stress. The MAPK pathway, extensively studied and linked with oxidative stress, is one such example. In the brain, MAPK pathway activation due to oxidative stress can result in increased cell apoptosis and inflammatory responses. Research has shown that inhibiting MAPK pathway activation can mitigate the oxidative stress-induced damage to neural cells, thereby safeguarding the brain from oxidative injury (Fan et al., 2019). In pulmonary diseases, the MAPK pathway also contributes to the modulation of oxidative stress. Findings reveal that activation of the MAPK pathway induced by oxidative stress escalates the production of inflammatory and fibrotic factors, thus aggravating lung damage (Maselli et al., 2002; Rezatabar et al., 2019).

To gain a more comprehensive understanding of the molecular mechanisms underpinning oxidative stress in brain–lung interactions, further investigation into other key molecular pathways, such as heme oxygenase-1 (HO-1) (Ryter and Choi, 2005) and SOD (Méthy et al., 2004), which also play substantial roles in oxidative stress responses, is essential. Additionally, given the intricacy of brain–lung interplay, future research endeavors should embrace an integrative methodology, incorporating transcriptomics, proteomics, and metabolomics, to thoroughly unravel the molecular intricacies of oxidative stress within the brain–lung nexus.

### 6.2 The potential of biomarkers in biomedical research

Biomarkers are instrumental as molecular or indicative targets for the assessment of specific biological processes or states of disease. In exploring the molecular mechanisms of oxidative stress and its brain–lung interactions, identifying suitable biomarkers for measuring the degree and impact of oxidative stress is of paramount importance.

In the context of AD, an increase in the levels of 8,12-iso-prostaglandin F2α (8,12-iso-PGF2α), indicative of enhanced lipid peroxidation within the body, has been noted (Praticò et al., 2000). This lipid peroxidation, a consequence of oxidative stress, is a marker for inflammation and cellular damage (Praticò et al., 2000), suggesting that 8,12-iso-PGF2α could be a potential biomarker for determining the extent of oxidative stress in the brain. In neurodegenerative diseases such as AD and PD, oxidative stress leads to increased lipid peroxidation chain reactions and protein nitration (Mythri et al., 2011; Butterfield, 2020). Therefore, the by-products of lipid peroxidation and protein nitration, like oxidized lipids and nitrotyrosine, are viewed as potential biomarkers for assessing the severity of oxidative stress in these neurodegenerative conditions.

In pulmonary diseases, oxidative protein products such as AOPPs and oxidized low-density lipoprotein (Ox-LDL) are considered potential biomarkers for gauging the level of oxidative stress (Shen et al., 2013; Gao et al., 2021). Studies have shown that AOPP levels are elevated in lung diseases like pneumonia and COPD, while Ox-LDL is linked to pulmonary fibrosis and airway inflammation (Shen et al., 2013).

Additionally, oxidative stress can lead to oxidative modifications in DNA and RNA. Biomarkers such as 8-hydroxyguanosine (8-OHdG) and 8-hydroxyadenine (8-OHdA) are extensively used to indicate oxidative damage in DNA and RNA (Valavanidis et al., 2009; Guo et al., 2020). The levels of these biomarkers, measurable in urine, blood, or tissue samples, serve to assess the degree of oxidative stress. However, their role in the interplay between the brain and lungs remains to be further elucidated.

## 7 Clinical applications of oxidative stress in brain–lung crosstalk

This section elaborates on the progress and trends of oxidative stress diagnostic tools, treatment methods, and future treatment strategies. Regarding oxidative stress diagnostic tools, emphasis is placed on the role of 8-OHdG and other related biomarkers in monitoring oxidative stress under different health conditions, as well as the development of advanced 8-OHdG measurement methods. In terms of innovative treatments for oxidative stress-related diseases, approaches such as enhancing NRF2 synthesis or inhibiting its degradation to alleviate oxidative stress, and utilizing non-toxic electron-affine substances to bind KEAP1 are mentioned. Additionally, innovative methods for treating cardiovascular diseases, neurodegenerative diseases, and glioblastoma are introduced. Lastly, future strategies for treating oxidative stress-related diseases are pointed out, such as applying machine learning to drug discovery, developing targeted drugs for specific diseases, and regulating the role of ROS in the wound healing process. Table 4 demonstrates summary of treatment strategies for oxidative stress-related diseases.

**TABLE 4:** Summary of treatment strategies for oxidative stress-related diseases.

Treatment strategy	Description	Application	Mechanism of action	References
Drug therapy	Traditional pharmacological approaches targeting specific symptoms or pathways of diseases.	Used in various oxidative stress-related diseases like neurodegenerative diseases, cardiovascular diseases, and cancer.	Often involves modulation of specific pathways or inhibition of pathological processes.	Firuzi et al., 2011; Zhang et al., 2011; Sá et al., 2017
Antioxidant therapy	Utilization of antioxidants like vitamin E, vitamin C, and beta-carotene to neutralize free radicals.	Commonly used in conditions with excessive oxidative stress, such as COPD and neurodegenerative diseases.	Directly reacts with free radicals to reduce oxidative stress and protect cells from damage.	Sorriento et al., 2018; Ashok et al., 2022
Mitochondrial targeting strategies	Strategies focusing on protecting or repairing mitochondrial function to reduce oxidative stress.	Emerging approach in diseases like neurodegenerative disorders where mitochondrial dysfunction is prominent.	Involves enhancing mitochondrial resilience to oxidative stress or repairing oxidative damage.	Cobley et al., 2018; Gonçalves and Romeiro, 2019
Machine learning in drug discovery	Integration of machine learning techniques to accelerate the identification and development of new therapeutic agents.	Applied in the discovery of novel drugs for treating oxidative stress-related conditions.	Improves efficiency and accuracy in identifying potential therapeutic compounds.	Vassalle et al., 2020
NRF2 synthesis enhancement	Enhancing the synthesis of NRF2 or inhibiting its degradation.	Investigated in various oxidative stress-related conditions for its antioxidant role.	NRF2 regulates the expression of antioxidant genes, thus protecting cells from oxidative damage.	Forman and Zhang, 2021
Antioxidant lipid peptides (via 3D-QSAR CoMSIA)	Identification of antioxidant lipid peptides through computational models.	A novel approach in antioxidant therapy, potentially applicable in various diseases.	Aims to optimize antioxidant properties of peptides for therapeutic use.	Forman and Zhang, 2021

### 7.1 Development of diagnostic tools

In recent years, oxidative stress biomarkers, particularly 8-OHdG, have received considerable attention due to their role in indicating oxidative DNA damage. This has been identified as a key biomarker of oxidative stress and has been shown to play an important role in the process of cancer development. Systematic reviews evaluating the assessment of 8-OHdG in various biological samples, including urine, have highlighted its utility in monitoring oxidative stress under different health conditions (Valavanidis et al., 2009; Graille et al., 2020). Further research has demonstrated the versatility of 8-OHdG in reflecting oxidative damage in occupational settings, particularly among workers exposed to nanomaterials (Omari Shekaftik and Nasirzadeh, 2021). Advanced methods for measuring 8-OHdG have been developed, such as the combination of terbium oxide nanoparticles and reduced graphene oxide, demonstrating progress in sensitive detection techniques for this biomarker (Manavalan et al., 2018).

Additionally, a non-invasive method for measuring another oxidative stress biomarker, 8-hydroxyguanine, in saliva, has been reported using high-performance liquid chromatography with electrochemical detection (HPLC-ECD). This method offers a simple and cost-effective option for monitoring oxidative stress (Kawai et al., 2018). In the context of specific diseases, MDA and 8-OHdG have been reported as reliable biomarkers of oxidative stress in ischemic stroke. The presence of these biomarkers in extracellular space, cerebrospinal fluid, and blood further validates their potential in diagnosing and monitoring neurodegenerative diseases (Tugasworo et al., 2023).

This new evidence on oxidative stress diagnostic tools, particularly focusing on 8-OHdG and related biomarkers, can provide important references for your review, offering comprehensive views on the current status and future prospects of oxidative stress diagnostics.

### 7.2 Innovation in treatment methods

In recent years, there have been numerous innovative treatment approaches in the field of oxidative stress-related diseases, aiming to alleviate oxidative stress through different mechanisms for the treatment or prevention of such diseases.

Nuclear factor erythroid 2-related factor 2, a key transcription factor within cells, protects cells from oxidative damage by regulating the expression of antioxidant genes. Studies have found that enhancing the synthesis of NRF2 or inhibiting its degradation could serve as a potential antioxidant treatment approach. Additionally, using non-toxic electrophiles to alkylate KEAP1 has also emerged as an important therapeutic means (Forman and Zhang, 2021). Furthermore, research has focused on how to prevent and treat diseases by targeting oxidative stress, including the utilization of innovative strategies such as identifying antioxidant lipid peptides through machine learning-integrated 3D-QSAR CoMSIA models (Vassalle et al., 2020).

In cardiovascular diseases, the imbalance of oxidative stress is one of the key factors leading to these diseases. It has been found that antioxidant treatment strategies, such as the use of antioxidants and drugs inhibiting oxidative stress generation, can serve as effective methods for treating these diseases (Pavlidis, 2022). Moreover, for neurodegenerative diseases like AD and PD, oxidative stress is considered a key driving factor in the pathological process. Treatment strategies involve the use of therapeutic agents like hydrogen sulfide to alleviate oxidative stress-induced neurodegeneration (Ienco et al., 2011). Additionally, in the treatment of glioblastoma, researchers are exploring potential therapeutic targets to modulate the extent of oxidative stress. This includes regulating the expression of key proteins like PKM2 to enhance resistance against oxidative stress (Liu S. et al., 2022).

These innovative treatment approaches demonstrate the rapid progress in the field of oxidative stress research, offering new perspectives and possibilities for the treatment of oxidative stress-related diseases. With further research, more innovative treatment strategies may emerge in the future.

### 7.3 Future strategies for oxidative stress-related diseases treatment

Researchers are exploring multiple novel strategies for the treatment of oxidative stress-related diseases in the future. These include applying machine learning techniques in drug discovery, specifically in the 3D-QSAR CoMSIA model for identifying antioxidant lipid peptides, which can improve the efficiency of antioxidant identification and design (Vassalle et al., 2020). Additionally, drugs targeting specific diseases such as Ebselen are being developed for inhibiting lung cancer cell growth through cell cycle arrest and cell death, as well as depleting glutathione (Vassalle et al., 2020). New anti-inflammatory and analgesic drugs such as LQFM291 have demonstrated their anti-inflammatory and analgesic effects, as well as good safety and toxicological properties (Vassalle et al., 2020). In intensive care units, the use of antioxidants has shown potential in counteracting oxidative stress (Vassalle et al., 2020). The regulation of ROS during wound healing process has also been shown to be an effective therapeutic pathway for promoting healing (Wang et al., 2023). Mitochondria-targeted therapeutic strategies, such as using light-sensitive NO-releasing molecules, are being studied for regulating mitochondrial oxidative stress (Yamada et al., 2020). Finally, modulating the role of oxidative stress in cancer and inflammation by controlling the production of endogenous ROS during tumor growth is a research focus (Fang et al., 2009). These studies demonstrate the innovative trends and potential strategies in the field of oxidative stress treatment, indicating the emergence of more innovative treatment methods in the future.

## 8 Conclusion

### 8.1 Summary of main findings

This comprehensive review has extensively explored the significance of oxidative stress in the brain–lung interaction, revealing its crucial role in various diseases. Biomarkers of oxidative stress, particularly 8-OHdG, have played important roles in the diagnosis and monitoring of this process. The medical community is striving to mitigate the impact of oxidative stress through various innovative therapeutic approaches, including drug therapies, machine learning techniques, and mitochondrial-targeted strategies. These findings offer new perspectives on the role of oxidative stress in brain and lung diseases and lay the foundation for future research and clinical interventions.

### 8.2 Discussion of future research directions

Future research should focus on further understanding the specific mechanisms of oxidative stress in brain–lung interactions and how to more effectively use this information to prevent and treat related diseases: (i) the impact of individual variations on oxidative stress responses should be considered, along with how to adjust treatment plans accordingly; (ii) the discovery and validation of novel biomarkers and the development of new drug delivery systems are also areas worth exploring; (iii) the application of machine learning and artificial intelligence technologies in drug discovery and disease prediction models is an important direction for future research; (iv) there is an urgent need for further research to dissect the molecular and cellular mechanisms that promote brain–lung interactions, with a special emphasis on the role of oxidative stress in mediating these interactions. This includes identifying key oxidative stress pathways common to brain and lung diseases; (v) the development of innovative therapeutic approaches targeting these oxidative stress pathways is expected to become a new treatment modality.

These strategies may include specially designed antioxidant therapies to alleviate oxidative stress in the brain and lungs, and the application of precision medicine approaches to tailor treatment methods according to the individual circumstances of patients. Exploring these areas will greatly promote our understanding of the complex relationship between brain and lung diseases, leading to more effective interventions.

### 8.3 Clinical and research implications of the review

This review emphasizes the clinical and research significance of oxidative stress in brain and lung diseases. A deeper understanding of oxidative stress can provide new strategies for early diagnosis and treatment of diseases. Furthermore, the mentioned treatment innovations, particularly advancements in drug development and the application of biomarkers, offer clinicians more treatment options and researchers new avenues to explore. These advancements not only help improve the quality of patients’ lives but also pave the way for new trends in scientific research.

## Author contributions

JK: Conceptualization, Data curation, Formal analysis, Funding acquisition, Investigation, Methodology, Project administration, Resources, Software, Supervision, Validation, Visualization, Writing – original draft, Writing – review & editing. RF: Conceptualization, Data curation, Formal analysis, Funding acquisition, Investigation, Methodology, Project administration, Resources, Software, Supervision, Validation, Visualization, Writing – original draft. YZ: Conceptualization, Data curation, Formal analysis, Funding acquisition, Investigation, Methodology, Project administration, Resources, Software, Supervision, Validation, Visualization, Writing – original draft. ZJ: Conceptualization, Data curation, Formal analysis, Funding acquisition, Investigation, Methodology, Project administration, Resources, Software, Supervision, Validation, Visualization, Writing – original draft. JZ: Conceptualization, Data curation, Formal analysis, Funding acquisition, Investigation, Methodology, Project administration, Resources, Software, Supervision, Validation, Visualization, Writing – review & editing. HP: Conceptualization, Data curation, Formal analysis, Funding acquisition, Investigation, Methodology, Project administration, Resources, Software, Supervision, Validation, Visualization, Writing – review & editing. QW: Conceptualization, Data curation, Formal analysis, Funding acquisition, Investigation, Methodology, Project administration, Resources, Software, Supervision, Validation, Visualization, Writing – review & editing.
